# Recent trends in airway management: we are not ready to give up fiberoptic endoscopy

**DOI:** 10.12688/f1000research.3829.1

**Published:** 2014-05-16

**Authors:** Davide Cattano, Rabail Chaudhry, Rashida Callender, Peter Killoran, Carin Hagberg

**Affiliations:** 1 Department of Anesthesiology, University of Texas Medical School at Houston, Houston, Texas, 77030, USA

## Abstract

The purpose of this correspondence is to discuss recent findings related to current trends in airway management and to discuss the utilization rates of video laryngoscopes *versus* traditional techniques in USA, UK, and Canada. To highlight the increased use of video laryngoscopes in difficult airway situations, data on the use of alternative airway devices at our institution collected from 2008 to 2010 are presented alongside the results of previously published surveys collected from 2002 to 2013.

## Correspondence

Education and research in anesthesia have increasingly focused on the management of difficult airways, leading to the development of new devices that are gradually becoming available and part of routine use across the globe. It is rather interesting to assess whether we have made much progress in using such devices over the past decade.

We read with great interest the letter ‘Should we really consider to lay down the Macintosh laryngoscope?’ ^1^, in which Merli G. *et al.* discuss the present and future roles of video laryngoscopes and the continued value of older instruments, *i.e.* the Macintosh direct laryngoscope. We agree with the authors that over the past two decades, a large number of airway devices have been introduced into clinical practice.

Data from the early 2000s suggest that, despite the widespread availability of newer airway equipment, traditional techniques (direct laryngoscopy, laryngeal mask airway (LMA), and flexible fiberoptic endoscopy) were the preferred techniques for intubation ( Table 1). Ezri *et al.* ^2^ reported in 2003 that US attending anesthesiologists preferably used flexible fiberoptic endoscopy (75%) for difficult airway management and preferred LMA (81%) in failed intubation/ventilation scenarios. Similarly, in 2004, fiberoptic endoscopy (64%) and some form of blind technique (26%) were used by anesthesiologists in the UK ^4^. In 2005, practitioners in Canada preferred fiberoptic endoscopy (34%) and direct laryngoscopy (48%) ^5^. In most surveys, lack of availability and training with newer equipment was of concern ^2– 5^.

**Table 1.** Outcomes of surveys completed regarding the preference of alternative airway management devices by geographical area and year completed.

Geographical area of survey	Year	Alternative device outcomes
Canada ^3^	2002	Fiberoptic (34%) and direct laryngoscopy (48%)
USA ^2^	2003	Fiberoptic (75%) for difficult airway management LMA (81%) in failed intubation/ventilation scenarios
UK, Oxford Region ^4^	2004	Fiberoptic (64%) and blind technique (26%)
Canada ^6^	2013	Video laryngoscope (90%)

We analyzed the utilization rates of alternative airway devices using data collected between 2008 and 2010 at our institution, the University of Texas Medical School at Houston, Memorial Hermann Hospital – Texas Medical Center ( Table 2).

**Table 2.** Alternative airway device usage rates and first attempt success rates at our institution, Memorial Hermann Hospital – Texas Medical Center at Houston, TX, USA: n, number of responders that prefer the use of a particular device for the majority of cases; usage rate, the percentage of responders that prefer the use of a particular device for the majority of cases; first attempt success rate, number of cases in which successful intubation was achieved in the first attempt.

Alternative airway device	(n)	Usage rate	First attempt success rate
Oral Fiberoptic (OFOI)	318	3.69%	92.5%
Glidescope ^®^ video laryngoscope (Verathon Inc, USA)	223	2.59%	95.5%
Storz C-MAC ^®^ video laryngoscope (Karl Storz, Germany)	154	1.79%	94.8%
Aintree intubation catheter (Cook Critical Care, USA)	106	1.23%	96.2%
Bougie	92	1.07%	85.9%
Nasal fiberoptic (NFOI)	92	1.07%	85.9%

The most commonly used alternative airway devices were oral fiberoptic intubation (OFOI), (n=318, usage rate=3.69%, first attempt success rate=92.5%), the Glidescope ^®^ video laryngoscopy system (Verathon Inc, USA), (n=223, usage rate=2.59%, first attempt success rate=95.5%), the Storz C-MAC ^®^ video laryngoscopy system (Karl Storz, Germany), (n=154, usage rate=1.79%, first attempt success rate=94.8%), the Aintree Intubation Catheter (Cook Critical Care, USA), (n=106, usage rate=1.23%, first attempt success rate=96.2%), bougie (n=92, usage rate=1.07%, first attempt success rate=95.7%) and nasal fiberoptic intubation (NFOI), (n=92, usage rate=1.07%, first attempt success rate=85.9%). Among these devices, OFOI and NFOI most likely required multiple intubation attempts, while the other devices had relatively high rates of success on the first intubation attempt.

When comparing our results with those obtained by Ezri *et al.* ^2^, the most striking difference is the increased use of video laryngoscopes. Ezri *et al.*, reported fiberoptic intubation and the LMA as the most popular in management of the difficult airway; no data was reported on the utilization rates of video laryngoscopes. The results of a similar survey completed by Canadian Anesthesiologists were recently presented at the Society of Airway Management Meeting 2013, where Mehta *et al.* ^6^ showed that the preferred alternative airway technique in difficult intubation situations was video laryngoscope. In a 2005 survey ^5^ the same authors found that the preferred devices were lighted stylet, bronchoscope, and intubating laryngeal mask airway ( Table 1).

There has been a rapid acceptance of video laryngoscopy as an important technique in the management of difficult airway situations. It is our opinion though, that while video laryngoscopy is preferred for ease of use and a faster learning curve, the technique of flexible fiberoptic endoscopy offers invaluable advantages: nasal and oral intubation, double lumen tube or bronchial blocker placement for thoracic surgery, therapeutic bronchoscopy, and it is preferred for awake technique intubation. The device versatility also makes it economical not to mention the greater value of education and training of future anesthesiologists.

## Supplementary materials

Pre-operative assessment form and post-operative evaluation used in the survey on alternative airway devices at the Memorial Hermann Hospital – Texas Medical Center at Houston, TX, USA.

Pre-operative Airway Assessment Form pdf file.

Post-operative Evaluation pdf file.
