# Supplementary material for: Recent trends in airway management: we are not ready to give up fiberoptic endoscopy
Source: F1000Res. 2014 May 16;3:114. [Version 1] doi: 10.12688/f1000research.3829.1 (PMC4118756; doi:10.12688/f1000research.3829.1)
Supplement: Peer review history

# Peer review: Referee response for version 1

**DOI:** 10.5256/f1000research.4102.r5326

**Published:** 2014-07-16

**Authors:** Ronald Pearl

**Affiliations:** 1 Department of Anesthesia, Stanford University Medical Center, Stanford, CA, USA

This report from one of the leading institutions in airway management confirms the rapid growth in the use of alternative airway devices, especially video laryngoscopes, but emphasizes that, on one hand, the majority of patients are still intubated using direct laryngoscopy, and, on the other hand, there remains an important role for fiberoptic intubation. The report documents a large number of available alternative airway devices but does not address the issues of how many different devices are required for the potential range of airway issues and how many devices can the standard practitioner be trained to use and maintain competency in their use.

I have read this submission. I believe that I have an appropriate level of expertise to confirm that it is of an acceptable scientific standard.

# Peer review: Referee response for version 1

**DOI:** 10.5256/f1000research.4102.r5327

**Published:** 2014-07-11

**Authors:** Suzanne Karan

**Affiliations:** 1 Department of Anesthesiology, University of Rochester Medical Center, Rochester, NY, USA

This article represents a growing body of research which will hopefully inform the appropriate education and training of our residents. The authors present data regarding the reported use of a variety of devices to manage the difficult airway. The venue of using *F1000Research* to quickly and more widely disseminate this information is highly valuable. The granularity of knowing user rate and first attempt success rate for these devices is a constructive addition that should be incorporated in future surveys to allow for comparison. With more data, it will be interesting to note whether the lower first attempt success for FOI (nasal or oral) stays the same or even drops compared with video laryngoscopy as the latter becomes more prevalently used and taught. The authors are commended in adding their research findings and their thoughtful opinions for review.

I have read this submission. I believe that I have an appropriate level of expertise to confirm that it is of an acceptable scientific standard.

# Peer review: Referee response for version 1

**DOI:** 10.5256/f1000research.4102.r4807

**Published:** 2014-05-30

**Authors:** Sairam Parthasarathy

**Affiliations:** 1 Pulmonary, Allergy, Critical Care, and Sleep Medicine, University of Arizona, Tucson, AZ, USA

Excellent and insightful information about approaches to intubation

I have read this submission. I believe that I have an appropriate level of expertise to confirm that it is of an acceptable scientific standard.
